# Determinants of Nurses’ Work Motivation in the Context of Sustainable Human Resource Management: A Study in a University Clinical Hospital in Poland

**DOI:** 10.3390/healthcare14172863

**Published:** 2026-09-05

**Authors:** Anna Rybarczyk-Szwajkowska, Anna Krakowiak, Agnieszka Strzelecka, Izabela Rydlewska-Liszkowska

**Affiliations:** 1 Department of Management and Logistics in Health Care, Faculty of Health Sciences, Medical University of Lodz, 90-419 Lodz, Poland; aniakrakowiak21@gmail.com; 2 Department of Econometrics and Statistics, Faculty of Management, Czestochowa University of Technology, 42-201 Częstochowa, Poland; agnieszka.strzelecka@pcz.pl; 3 Department of Medical Insurance and Health Care Financing, Faculty of Health Sciences, Medical University of Lodz, 90-419 Lodz, Poland; izabela.rydlewska-liszkowska@umed.lodz.pl

**Keywords:** Sustainable Human Resource Management, nurses, work motivation, motivating factors, demotivating factors, financial incentives, non-monetary incentives, non-material motivation, nursing staff, clinical hospital, healthcare management

## Abstract

**Background/Objectives:** Sustainable Human Resource Management in healthcare requires the identification of factors that support motivation, well-being, and employment sustainability in nursing staff. The aim of this study was to assess motivating and demotivating factors related to nurses’ work motivation in a Polish university clinical hospital and to examine their associations with selected sociodemographic and occupational characteristics in order to identify Sustainable HRM practices that may be relevant to staff retention, professional resilience, and long-term workforce sustainability. **Methods:** The study was conducted among 300 nurses using a diagnostic survey method between October 2023 and March 2024. Associations between variables were examined using the χ^2^ test of independence and Cramér’s V. **Results:** Salary level was the most important financial motivating factor in the nurses’ opinions. With regard to non-wage factors, the opportunity to improve qualifications at the employer’s expense was predominant. Friendly team relationships were identified as the key non-material motivating factor. The most frequently indicated demotivators were low salary, low occupational status, poor relationships with supervisors or team members, and limited autonomy. Significant associations were observed between financial motivating factors and age, professional experience, employment type, and salary level. Associations for indirect material, non-material, and demotivating factors were less consistent and generally weak. **Conclusions:** Effective nursing workforce management should combine fair remuneration with opportunities for professional development and measures that support positive workplace relationships and organizational culture. Interpreted within the Sustainable HRM framework, these findings may inform practices intended to support staff retention, professional resilience, and long-term workforce sustainability.

## 1. Introduction

The notion Sustainable Human Resource Management (Sustainable HRM) does not exclusively refer to short-term efficiency-oriented human resource management but goes beyond that. It also emphasizes the long-term preservation, development, and regeneration of human resources [1,2]. The concept integrates economic, human, social, and environmental aspects [3], while common-good-oriented approaches additionally point out the responsibility of HRM towards employees and wider stakeholder groups [4].

This perspective particularly applies to healthcare organizations, where service continuity depends on a healthy, competent, and stable workforce. At the organizational level, Sustainable HRM is associated with the implementation of SDG 3 (Good Health and Well-being) and SDG 8 (Decent Work and Economic Growth) [5,6]. Sustainable HRM may be operationalized through long-term orientation, employee care, development opportunities, participation, fairness, and work design that protects employee health [7]. Such practices combining organizational performance with employee well-being [8] may reduce work-related stress and burnout [9]. Studies conducted in healthcare and care settings have also associated Sustainable HRM practices with employee resilience, happiness at work, and broader well-being [10].

Building on this organizational perspective, the 2030 Agenda provides a broader policy framework for understanding why healthcare workforce sustainability is integral to health-system performance. Nursing is most directly connected with SDG 3 and SDG 8 through the delivery of healthcare and the need for decent and secure work. In the broader literature, nursing is also discussed in relation to SDG 5 because it is a predominantly female profession, and in relation to SDG 17 through collaboration among professional, organizational, governmental, and international stakeholders [5,6,11]. However, the present study did not examine gender inequalities or gender-based differences; therefore, SDG 5 is considered only as part of the broader theoretical context and not as an empirical outcome of this study. These interdependencies position nurses not only as providers of clinical care but also as contributors to health promotion, education, service coordination, and the implementation of system-level change [6,11].

The SDGs play a role in human resource management. They do not merely constitute an external policy agenda, but a framework combining employment quality, workforce capacity, and the long-term ability of healthcare organizations to deliver safe services [5,6].

In Poland, Sustainable HRM research has included analysis of the health consequences of fixed-term employment [12] and the relationship between the well-being of employees, their development, retention, engagement, and satisfaction [13]. Studies on nurses have also demonstrated the importance of remuneration and other motivators of job satisfaction [14], as well as the role of workload, burnout, promotion prospects, and the work environment in the context of nurses’ intention to quit their job [15]. Nevertheless, these lines of research are insufficiently integrated, particularly in the context of university clinical hospitals.

The aim of this study was to assess motivating and demotivating factors related to nurses’ work motivation in a Polish university clinical hospital and to analyze their associations with selected sociodemographic and occupational characteristics in order to identify Sustainable HRM practices that may be relevant to staff retention, professional resilience, and long-term workforce sustainability.

The study was guided by the following research questions:

RQ1. To what extent are nurses motivated by financial motivating factors, identified as direct financial incentives; non-wage motivating factors, identified as indirect monetary incentives; non-material factors, identified as psychological incentives, considering differences regarding age, work seniority, type of employment, and level of remuneration?

RQ2. Does the perception of demotivating factors differ depending on nurses’ age, work seniority, type of employment, and level of remuneration?

RQ3. Considering the research results, what Sustainable HRM practices can be proposed to support nursing staff retention, professional resilience, and long-term workforce sustainability?

## 2. Sustainable Human Resource Management and Nurses’ Motivation: Theoretical and Organizational Background

### 2.1. Sustainable Human Resource Management in Healthcare: Conceptual Foundations and Implementation

Sustainable Human Resource Management can be defined as a system of planned and mutually reinforcing human resource policies and practices that enables an organization to achieve its objectives while retaining, developing, and regenerating its human resource base in the long run [1,2,3]. In contrast to conventional approaches, according to Sustainable HRM, organizational outcomes should not be achieved through the excessive depletion of employees’ physical, psychological, or professional resources. Hence, Sustainable HRM evaluates not only economic aspects but also employee health, well-being, employability, social responsibility, and continuity of employment [1,2].

The literature on Sustainable HRM encompasses several perspectives, including socially responsible HRM, Green HRM, triple-bottom-line HRM, and common-good-oriented HRM [3,4]. The present study has primarily adopted a human and social sustainability perspective. Accordingly, sustainability is understood not mainly as the promotion of employees’ environmental behaviors, but as the organization’s long-term capacity to maintain a healthy, competent, motivated, and sufficiently stable workforce [1,3,4]. This distinction is particularly important in healthcare organizations, where the continuity and quality of services depend directly on the availability, competence, and well-being of healthcare professionals. Short-term efficiency pressures, intensive workloads, shift work, high levels of responsibility, and emotional demands may temporarily support service continuity, but they may also contribute to work-related stress, emotional exhaustion, burnout, and reduced job satisfaction [8,9,16]. From the Sustainable HRM perspective, immediate operational requirements should therefore be balanced with the long-term protection, development, and regeneration of employees’ resources [1,2,7]. The Sustainable Development Goals connect workforce practices with broader health and social outcomes. SDG 3 includes the development and retention of the healthcare workforce as a condition for access to healthcare, while SDG 8 emphasizes productive employment, decent work, and the protection of employees’ rights. The broader nursing literature also links the profession with SDG 5 because equal access to leadership and professional advancement is relevant in a predominantly female profession, and with SDG 17 because partnerships are required to coordinate workforce policy, education, research, and service delivery [5,6,11]. However, gender inequalities were not directly measured in the present study. Accordingly, the empirical interpretation of the findings focuses primarily on SDG 3 and SDG 8. The literature on nursing also indicates that engagement with the SDGs requires institutional capacity rather than reliance on individual commitment alone. Healthcare organizations should translate the SDGs into workforce policies, professional development, leadership opportunities, and mechanisms that enable nurses to participate in organizational improvement [6,11,17,18,19].

Sustainable HRM in healthcare is not implemented through a single HR practice. It requires a coherent system of mutually reinforcing policies and practices covering different stages and employment contracts. These practices can be analytically categorized into five interrelated domains [7,8,9,12,13,16].

The first domain concerns health-protective work design, including adequate staffing, manageable workloads, predictable scheduling, opportunities for rest and recovery, psychological support, and the prevention of excessive occupational stress. Evidence from hospital settings also indicates that HR systems and working conditions may be associated with emotional exhaustion, work engagement, and job satisfaction [16].

The second domain comprises fair and stable employment practices, including transparent remuneration, employment security, procedural fairness, equal access to rewards and benefits, and types of employment that do not burden employees with a disproportionate amount of organizational risk. Research conducted in the Polish environment also indicates that fixed-term employment may have an adverse effect on employees’ health and their perception of security [12].

The third domain refers to the development of competencies and long-term employability. It includes access to training, mentoring, career development, professional advancement, knowledge sharing, and the adaptation of development opportunities to employees at different stages of their career and lives [1,7]. Polish research has also demonstrated relationships among development opportunities, employee well-being, their engagement, retention, and job satisfaction [13].

The fourth domain involves supportive leadership and employee participation, including respectful communication, professional recognition, autonomy, involvement in decision-making that affects work, constructive feedback, and effective conflict management. In hospital settings, supportive HR practices may also contribute to work engagement and job satisfaction while reducing emotional exhaustion [16].

The fifth domain relates to employee retention and workforce continuity. It includes age-sensitive HR practices, succession planning, flexible work arrangements, internal mobility, and career continuity, as well as the systematic monitoring of absenteeism, job satisfaction, employee well-being, burnout risk, and intentions to resign. Retention should not be understood as an isolated HR outcome but as a result of the interaction among employment stability, development opportunities, employee well-being, organizational support, and the quality of the working environment [8,12,13,16].

These five domains should be treated as components of an integrated HR system. For example, professional development opportunities may have limited motivating value when employees simultaneously experience excessive workloads, employment insecurity, insufficient recognition, or a lack of managerial support. Similarly, monetary incentives alone may not ensure long-term retention if the employees do not experience fair treatment, autonomy, supportive relationships, psychological safety, and adequate opportunities for recovery in their work environment [7,8,9,12,13,16]. In university clinical hospitals, Sustainable HRM is not exclusively identified with rendering clinical services. These organizations integrate patient care with teaching and research activities. Therefore, they depend on a stable and highly qualified workforce which must be capable of contributing to all three functions [20].

### 2.2. Nurses’ Motivation, Job Satisfaction, and Workforce Sustainability

Previous studies indicate that nurses’ motivation and job satisfaction are multidimensional and cannot be explained solely by remuneration. Burnout and reduced work engagement are highly common problems among nursing staff [21], and nurse burnout puts patient safety at risk [22]. Evidence from systematic reviews also implies that there is a link between burnout and demanding healthcare conditions, as well as adverse psychological outcomes [23,24]. However, interventions that strengthen individual and organizational resources may reduce this burnout [25]. Health emergencies and prolonged exposure to occupational stressors further intensify the risk of burnout [26], including among Polish healthcare workers [27]. At the same time, intrinsic and extrinsic motivation may perform different functions: intrinsic motivation can support engagement, whereas external factors may be particularly important in job selection or resigning from work [28]. The international literature [29] and research on the relationship between job satisfaction and burnout [30] support the need to conduct a joint analysis of motivators and demotivators.

Financial compensation is important, particularly when employees perceive a discrepancy between workload, responsibility, qualifications, and remuneration. However, non-material factors—including recognition, autonomy, leadership quality, team relations, professional development, meaningful work, and participation in decision-making—also play an important role in maintaining motivation and organizational commitment. Consequently, the same factor may serve a different function depending on whether the studied outcome is daily work motivation, job satisfaction, burnout, or intention to resign from work [7,14,15].

### 2.3. Polish University Clinical Hospitals as an Organizational Context

The Polish healthcare system has been facing understaffing and the aging of nursing personnel. National statistics and professional workforce analyses indicate that maintaining the size and continuity of the nursing workforce is an increasingly important management challenge [31,32]. Polish studies have provided valuable evidence on the influence of the work environment on patient safety [33], occupational burnout [34], and the relationship between nurses’ working conditions and professional quality of life [35].

At the same time, Sustainable HRM research conducted in Poland has addressed employee development [36], perceptions of Sustainable HRM and personal risk [37], employment stability and health [12], as well as relationships among well-being, development, retention, engagement, and satisfaction [13]. Researchers studying nursing personnel have also analyzed job satisfaction and remuneration [14] and the intention to resign from work [15]. All these studies provide relevant evidence on employee health, well-being, motivation, job satisfaction, and workforce retention across distinct research aspects and organizational settings.

The university clinical hospital constitutes an underexplored organizational context. Such hospitals require a stable and highly qualified workforce which is capable of providing complex care, supervising students and junior professionals, participating in interdisciplinary teams, and contributing to research activities [20].

Table 1 synthesizes selected studies and shows how the present study is positioned in relation to their theoretical perspectives, organizational contexts, variables, and addressed research gaps.

### 2.4. Research Gaps and Theoretical Contribution

As summarized in Table 1, previous studies have provided important but fragmented evidence concerning Sustainable HRM. Three interrelated research gaps can therefore be identified. Firstly, previous studies have often analyzed occupational stress, burnout, job satisfaction, motivation, or intention to resign from work as separate research areas, while fewer studies have integrated motivating and demotivating factors within a single analytical and interpretative framework. Secondly, the Sustainable HRM concept has rarely been used to translate nurses’ motivating experiences into specific retention-oriented, development-oriented, and health-protective HR practices in the Polish healthcare context. Thirdly, university clinical hospitals are still unexplored despite their combined clinical, teaching, and research functions.

The theoretical contribution of this study involves linking the micro-level perspective of work motivation with the meso-level perspective of organizational HR practices. In this study, motivation is not treated solely as an individual psychological state but as an employee-related outcome that may be associated with the design and implementation of HR policies and practices. The study therefore offers a context-specific interpretation of Sustainable HRM by showing how the identified motivating and demotivating factors may inform practices intended to support and retain nursing personnel.

## 3. Materials and Methods

### 3.1. Study Design and Setting

A cross-sectional questionnaire study was conducted between October 2023 and March 2024 in a university clinical hospital in Poland. The hospital was purposively selected because of its status as a university clinical hospital, combining clinical, teaching, and research functions. This organizational context was considered particularly relevant to the analysis of nurses’ work motivation from the Sustainable HRM perspective.

### 3.2. Study Population, Recruitment and Eligibility Criteria

The study population comprised all nurses employed at the university clinical hospital involved in the study and actively performing nursing duties during the data collection period. All 645 eligible nurses were invited to participate through the hospital’s internal e-mail system. The invitation explained the purpose of the study, the voluntary and anonymous nature of participation, the confidentiality of data handling, and the exclusive use of the findings for scientific purposes. It also included a link to the electronic questionnaire and information about the availability of the paper version.

The designated researcher who provided access to the paper questionnaire was employed at the hospital but was not a supervisor of the participants and was not involved in evaluating their work. Eligibility criteria included employment as a nurse at the participating hospital, active performance of nursing duties during the study period, and voluntary participation. No additional exclusion criteria were applied. Completion and return of the questionnaire were regarded as informed consent.

Of the 645 nurses invited, 300 returned questionnaires that were included in the analysis, which corresponded to a response rate of 46.5%.

### 3.3. Survey Questionnaire

An author-designed survey questionnaire comprising 24 items was used as the research instrument. The first section assessed factors related to professional motivation, reasons for choosing the nursing profession, and symptoms of occupational burnout. Based on a review of the literature [28,38,39,40], motivating factors were classified into three categories: (1) financial motivating factors (direct monetary incentives), (2) non-wage motivating factors (indirect material incentives), and (3) non-material motivating factors (psychological incentives). For each category of motivating factors, respondents were asked to select the one factor that they considered the most important. Each participant therefore selected one financial factor, one non-wage factor, and one non-material factor. Respondents also selected one primary demotivating factor. Multiple responses within the same category were not permitted, and the factors were not rated on a Likert scale or ranked. The resulting variables were categorical and mutually exclusive within each analysis.

The second section presented demotivating factors, while the final section collected sociodemographic and occupational data. The survey was administered in both paper and electronic formats. An anonymized version of the questionnaire is provided as Supplementary File S1.

### 3.4. Data Analysis

To analyze associations between selected sociodemographic and occupational variables—i.e., age, work seniority, type of employment, and remuneration level—and the perception of motivating factors, including financial, non-wage, and non-material factors, as well as demotivating factors, the χ^2^ test of independence was applied. The strength of the observed associations was assessed using Cramér’s V, which provides a standardized measure for contingency tables of different dimensions. Cramér’s V quantifies the strength, but not the direction, of an association between categorical variables. All statistical analyses were performed using Microsoft Excel 2019 (Microsoft Corporation, Redmond, WA, USA).

### 3.5. Ethical Issues

The study was conducted in accordance with ethical principles regarding research involving human subjects, in compliance with the Declaration of Helsinki of the World Medical Association (WMA) and applicable national regulations concerning the protection of research participants. The hospital administration provided formal approval for the research project and for conducting the survey among employees.

Each participant was informed that participation in the study was voluntary and that the collected data would be fully anonymous and confidential. The introduction to the questionnaire outlined the study’s purpose and the scope of data processing and assured participants that the data would be used exclusively for scientific purposes, in accordance with the principles of data minimization and respect for privacy. Participation in the study was considered equivalent to giving informed consent. The study did not involve procedures that interfered with the physical or psychological integrity of the participants, and the collected data did not allow for the identification of individuals participating in the project.

## 4. Results

### 4.1. Socio-Demographic and Professional Characteristics

The sociodemographic and occupational characteristics of the study sample are summarized in Table 2. The study included 300 nurses, most of whom were women (92.7%). The largest proportion of respondents were aged 30–49 years (46.0%), had up to 10 years of work seniority (44.3%), and were employed under permanent employment contracts (64.7%). More than half of the participants reported monthly earnings between PLN 6600 and 9500 (54.7%), while the majority had a Master’s degree (65.7%).

The mean age of the respondents was 39.28 years (SD = 11.41), and the mean length of professional experience was 14.19 years (SD = 9.78). The mean reported salary was 6773.17 PLN (SD = 1184.04), with a median of PLN 6755.56.

Salary level (54.0%; *n* = 162) was the most frequently indicated financial motivating factor (Table 3). It clearly predominated over other monetary incentives, including performance-related bonuses (25.3%; *n* = 76). Among non-wage motivating factors, the opportunity to improve qualifications at the employer’s expense was the most commonly selected factor (79.3%; *n* = 238), whereas the remaining categories were indicated substantially less often. With regard to non-material motivating factors, friendly team relationships accounted for the highest proportion of responses (57.7%; *n* = 173), followed by flexible working hours (27.7%; *n* = 83).

The distribution of demotivating factors indicates that a low salary was the predominant factor reducing work motivation among nurses, reported by 48.7% of respondents. Low occupational status was identified by 22.3% of participants, followed by poor relationships with supervisors or the team (15.7%) and limited autonomy or decision-making power (13.3%) (Table 4).

### 4.2. Analysis of Financial Motivating Factors (Direct Monetary Incentives) According to Age, Work Experience, Type of Employment, and Salary Level

All analyzed variables—age, work experience, type of employment, and salary level—showed statistically significant associations with financial motivating factors (Table 5). However, all associations were weak, with Cramér’s V values ranging from 0.158 to 0.194. The results indicate differences in the distribution of responses across the analyzed groups but do not establish the direction of the associations.

At the 0.01 significance level, statistically significant associations with financial motivating factors were observed for the respondents’ age (χ^2^ = 22.505; *p* = 0.01) and work experience (χ^2^ = 20.162; *p* = 0.01). Cramér’s V values for these variables were 0.194 and 0.183, respectively, indicating weak associations. Cramér’s V measures the strength of association between categorical variables and does not indicate its direction.

Salary level was also statistically significantly associated with financial motivating factors (χ^2^ = 14.906; *p* = 0.05; Cramér’s V = 0.158). The association was weak. This result indicates an association but does not establish its direction or support causal conclusions regarding salary level and expectations concerning financial rewards.

The type of employment was statistically significantly associated with financial motivating factors (χ^2^ = 10.036; *p* = 0.02; Cramér’s V = 0.183). The association was weak. The analysis does not indicate its direction and does not support a causal interpretation of the relationship between employment type and financial motivation.

### 4.3. Analysis of Non-Wage Factors (Indirect Material Incentives) According to Age, Work Experience, Type of Employment, and Salary Level

The analysis examined associations between age, work experience, type of employment, salary level, and the selected non-wage motivating factors (Table 6). Age, work experience, and salary level were not statistically significantly associated with the selected non-wage motivating factors. These results indicate that no statistically significant associations were detected for these variables in the present sample; they do not demonstrate equivalence between groups.

For statistically insignificant variables the interpretation of association measures should be approached with caution. These coefficients were calculated primarily to maintain consistency across the analyzed sets of variables and to indicate the level at which the χ^2^ test of independence would suggest a statistically significant relationship.

The only variable significantly associated with non-wage motivating factors was the type of employment (χ^2^ = 14.657; *p* = 0.01; Cramér’s V = 0.221). The association was weak. The analysis confirms the presence of an association but does not determine which employment group assigned greater importance to particular non-wage motivating factors.

### 4.4. Analysis of Non-Material Factors According to Age, Work Experience, Type of Employment, and Salary Level

The analysis of non-material factors, such as recognition, professional prestige, autonomy, relationships with patients, and professional ethics, complemented the assessment of factors related to nurses’ work motivation. The third stage of the analysis examined associations between selected sociodemographic and occupational variables and the importance attributed to non-material motivating factors.

Based on the results presented in Table 7, work experience showed a statistically significant association with non-material motivation (χ^2^ = 13.732; *p* = 0.01; Cramér’s V = 0.151). The association was weak and does not establish that the importance of professional recognition, prestige, or a sense of mission increases with professional experience.

Age was also statistically significantly associated with non-material motivating factors (χ^2^ = 10.157; *p* = 0.05; Cramér’s V = 0.130). By contrast, the type of employment was not statistically significantly associated with non-material motivating factors (χ^2^ = 5.630; *p* = 0.06; Cramér’s V = 0.137). Salary level was also not significantly associated with these factors (χ^2^ = 2.409; *p* = 0.70; Cramér’s V = 0.063).

### 4.5. Analysis of Demotivating Factors According to Age, Work Experience, Type of Employment, and Salary Level

To complement the analysis, demotivating factors were also examined (Table 8). In addition to the previously analyzed financial, non-wage, and non-material motivating factors, the analysis considered selected demotivators, including poor relationships, psychological burden, low occupational status, and the shifting of responsibility.

Among the analyzed variables, work experience was the only characteristic significantly associated with perceived demotivation (χ^2^ = 14.755; *p* = 0.05; Cramér’s V = 0.157). The association was weak. Cramér’s V quantifies the strength of association but does not indicate its direction; therefore, no conclusion can be drawn that nurses’ sensitivity to particular demotivating factors increases or decreases with work experience.

Age was not statistically significantly associated with demotivating factors (*p* = 0.10). Therefore, the analysis does not provide sufficient evidence that age is associated with differences in the perception of demotivating factors.

No statistically significant associations were found between salary level or the type of employment and the perception of demotivating factors. These results indicate that no statistically significant associations were detected in this sample; they should not be interpreted as evidence that perceptions were identical across groups or independent of pay conditions and employment type.

## 5. Discussion

### 5.1. Principal Findings and Comparison with Previous Research

The results of this study indicate that nursing staff motivation is an important element of Sustainable Human Resource Management in healthcare. In the context of achieving the Sustainable Development Goals (SDGs), particularly SDG 3—Good Health and Well-being—and SDG 8—Decent Work and Economic Growth—it is particularly important to identify factors that support employment stability, staff retention, quality of care, and patient safety. The study revealed statistically significant associations between selected sociodemographic and occupational characteristics and the financial motivation of nursing staff. The strongest association was observed for age (Cramér’s V = 0.194), followed by the length of professional experience (Cramér’s V = 0.183); both associations were weak. Cramér’s V measures the strength of association between categorical variables and does not indicate its direction. Previous studies suggest that older generations of nurses more often value stable salary components and economic security, whereas younger staff more often associate remuneration with immediate gratification, flexibility, professional development, and a faster return on investment in education and competencies [41,42]. However, the present analysis confirms only the existence of an association and does not determine its direction.

Furthermore, remuneration should be considered in its legal and regulatory context. The Act of 8 June 2017, on the method of determining the minimum base salary for certain employees working in healthcare facilities in Poland, introduced mandatory work components, thereby making base salary a more regulated component of the remuneration system [43]. This regulatory context may be relevant when interpreting the role of remuneration as a motivating factor, but the present study does not establish causal effects of the legislation. The type of employment was significantly but weakly associated with financial motivating factors. The analysis did not determine the direction of this association and therefore does not indicate which employment group placed greater emphasis on particular monetary incentives.

Age and length of professional experience were not statistically significantly associated with the selected non-wage factors. This means that no statistically significant differences in response distributions were detected for these variables in the present sample; it does not demonstrate that the underlying needs were universal. The type of employment was significantly associated with non-wage factors, whereas salary level was not significantly associated with non-material motivating factors. These findings describe statistical associations or their absence and should not be interpreted as causal effects. Previous research suggests that remuneration alone may be insufficient to support engagement, satisfaction, and retention [44], while autonomy, responsibility, and professional recognition may be relevant to occupational commitment [45,46].

Low salary was the most frequently selected demotivating factor, while low occupational status, poor workplace relationships, and limited autonomy were also reported. Professional experience was weakly associated with the selected demotivating factors, whereas age, employment type, and salary level were not statistically significantly associated. The absence of statistical significance should not be interpreted as evidence that pay or employment conditions are unimportant. Because the study was cross-sectional, no causal conclusions can be drawn regarding employee characteristics and demotivation. Psychological strain, workload, staffing shortages, organizational conflicts, and lack of security are discussed only as contextual factors identified in previous research [47,48,49,50], not as outcomes established by the present analysis.

Taken together, these findings reinforce the need for changes in healthcare systems aimed at improving effectiveness, accessibility, and quality of care. Healthcare workers are central to these changes, with nursing staff playing a particularly significant role [5,6,11]. The multifaceted role of nurses involves not only clinical competencies but also educational, organizational, social, and environmental potential. The implementation of the Sustainable Development Goals can improve the quality, accessibility, and outcomes of health and nursing care when these goals are integrated into health policy, nursing education, and daily clinical practice. Deepa et al. [51] highlight a positive relationship between SDG implementation and the quality of care, access to services, and patient satisfaction, particularly in nursing practice. Similarly, Sensor et al. [6] discuss nursing in relation to SDG 5, SDG 8, and SDG 17. However, because the present study did not measure gender inequalities or examine gender-based differences, its findings should not be interpreted as empirical evidence concerning SDG 5. The interpretation of the present results is therefore focused on SDG 3 and SDG 8, which are directly related to the analyzed issues of work motivation, remuneration, working conditions, professional development, and nursing workforce sustainability.

### 5.2. Academic Implications

This study contributes to the Sustainable HRM literature by relating financial, non-wage, and non-material motivators, together with demotivating factors, to organizational HR practices in a university clinical hospital. The findings support the view that motivators and demotivators should not be treated as simple opposites: remuneration may be perceived both as an incentive and as a condition of fairness. The study also provides context-specific evidence from a Polish university clinical hospital, but the low Cramér’s V values call for caution when categorizing nurses by age, professional experience, or employment status.

### 5.3. Practical Implications

At the organizational and system levels, it is important to consider how nursing staff can be involved in the implementation of sustainable development goals in healthcare facilities, local initiatives, and national and global policies. In this context, strategies intended to strengthen the nursing workforce may include education, improved working conditions, satisfactory remuneration, leadership development, and the recognition of the nurses roles in achieving the Sustainable Development Goals in healthcare [17,18,19]. Nursing staff management strategies should therefore consider compensation, working conditions, professional development, autonomy, recognition, mental well-being, and organizational culture. From the perspective of SDG 8, the findings may inform wage policies intended to support economic security, perceived fairness, and staff retention. Directive (EU) 2023/970 on pay transparency provides a broader regulatory context for considering remuneration equity in healthcare institutions [52]. The findings concerning non-wage expectations may also be considered in relation to Directive (EU) 2019/1158 on work–life balance [53]. The observed association between the employment type and non-wage factors may be considered in the broader regulatory context of Directive (EU) 2019/1152 on transparent and predictable working conditions [54]. However, the present analysis does not establish causal effects of these directives or determine which employment group values particular incentives more strongly.

### 5.4. Limitations and Future Research

A limitation of the study is that it was conducted at a single teaching hospital in central Poland. The specific nature of this facility, including the complexity of care, its educational function, and its organizational structure, may limit the generalizability of the findings to non-university healthcare settings. The results should therefore be interpreted in the context of the hospital under study. The cross-sectional design and self-reported data do not permit causal conclusions and may be affected by response bias. The response rate was 46.5%. A formal non-response bias analysis was not performed because individual-level sociodemographic and occupational data were unavailable for the 345 nurses who did not participate. Therefore, respondents and non-respondents could not be compared with respect to age, professional experience, ward or department, or other relevant characteristics. Consequently, systematic differences between respondents and non-respondents cannot be excluded, which may limit the representativeness and generalizability of the findings. In addition, the author-designed questionnaire and broad response categories may have limited the precision with which individual motivating and demotivating constructs were measured. Future research should include different types of healthcare facilities and regions, use validated multi-item measures, and apply longitudinal or intervention designs to examine temporal and potentially causal relationships.

## 6. Conclusions

Work motivation in the studied university clinical hospital as perceived by nurses, was multidimensional. Salary level was the most frequently selected financial motivator, professional training funded by the employer was the most common indirect material motivator, and friendly team relationships were the most frequently considered non-material motivator. Low salary was the leading demotivating factor.

Financial motivating factors were associated with age, professional experience, employment type, and salary level. The patterns regarding indirect material, non-material, and demotivating factors were less consistent, and all statistically significant associations were weak. These findings do not support a rigid categorization of nursing staff but suggest that remuneration, professional development, employment conditions, and relational aspects of work should be considered jointly.

From a Sustainable HRM perspective, nursing workforce management should combine fair remuneration with access to professional development, supportive workplace relationships, autonomy, and recognition. The findings are relevant to SDG 3 and SDG 8 because the well-being of employees, decent working conditions, and the continuity of healthcare services are interrelated. However, the cross-sectional and single-center design requires cautious interpretation, and the findings should be confirmed in multicenter studies using validated measures and multivariable analyses.

## Figures and Tables

**Table 1:** Synthesis of selected studies on Sustainable Human Resource Management and positioning of the present study.

Study	Main Focus	Key Contribution or Finding	Research Gap in the Present Study
Kramar (2014) [1]	Foundations of Sustainable HRM	Human resource management (HRM) should consider long-term human, social, and organizational outcomes rather than economic performance alone.	Provides a theoretical foundation but does not operationalize Sustainable HRM in relation to nurses’ motivation or healthcare workforce continuity.
Stankevičiūtė and Savanevičienė (2018) [7]	Core characteristics of Sustainable HRM	Identifies long-term orientation, employee care, development, participation, fairness, and employee-friendly work design as central characteristics of Sustainable HRM.	Does not study how these practices relate to different categories of nurses’ motivators and demotivators.
Staniškienė et al. (2026) [10]	Sustainable HRM, employee resilience, and happiness at work	Sustainable HRM was positively associated with employee resilience and happiness at work; resilience partially mediated the relationship between Sustainable HRM and happiness at work.	Did not focus specifically on nurses and did not analyze financial, indirect material, and non-material motivators together with demotivating factors.
Piwowar-Sulej and Bąk-Grabowska (2020) [12]	Employment for a definite period of time employee health, and Sustainable HRM	Fixed-term employment may have adverse consequences for employee health and employment sustainability.	Does not examine healthcare employees or the motivating structure of nursing personnel.
Sypniewska et al. (2023) [13]	Employee well-being, development, retention, engagement, and satisfaction	Development-, well-being-, and retention-related practices were associated with employee engagement and satisfaction.	Used a cross-sectoral sample and did not examine nurses, demotivators, or university hospitals.
Kunecka (2018) [14]	Job satisfaction, remuneration, rewards, and professional advancement	Remuneration and other motivators significantly influenced nurses’ job satisfaction.	Did not combine financial, indirect material, and non-material motivators with demotivating factors or a Sustainable HRM interpretation.
Malinowska-Lipień et al. (2023) [15]	Work environment, burnout, job satisfaction, and intention to quit the job	Workload, emotional exhaustion, remuneration, promotion opportunities, and job satisfaction were related to nurses’ intention to resign from work.	Did not differentiate the structure of motivating factors or translate the findings into Sustainable HRM practice domains.
Mihail and Kloutsiniotis (2016) [16]	HR systems, engagement, emotional exhaustion, and job satisfaction	HR systems were associated with hospital employees’ work-related well-being, engagement, and emotional exhaustion.	The sample was not nurse-specific, the Polish context was not studied, and the study was not explicitly formulated within a Sustainable HRM framework.
Present study	Financial, indirect material, and non-material motivators; demotivating factors;	This study examines relative importance of motivators and demotivators and their associations with age, professional experience, employment type, and salary level.	Determination the role of motivating and demotivating factors in shaping work motivation among nurses employed in a Polish university teaching hospital and analyzing the associations between these factors and selected sociodemographic and professional characteristics; identifying Sustainable Human Resource Management practices that may support staff retention, professional resilience and the long-term stability of human resources.

Abbreviations: HRM—human resource management. Sustainable HRM—Sustainable Human Resource Management. Source: Authors’ synthesis based on the studies cited in the table.

**Table 2:** Characteristics of the Respondents.

Variable	Category	*n*	%
Age	23–29	87	29.0
	30–49	138	46.0
	50–63	75	25.0
Gender	Female	278	92.7
	Male	22	7.3
Work seniority	0–10 years	133	44.3
	11–20 years	79	26.3
	More than 20 years	88	29.3
Type of employment	Permanent employment contract	194	64.7
	Fixed-term employment contract	106	35.3
Salary	3500–5500 PLN	31	10.3
	5600–6500 PLN	105	35.0
	6600–9500 PLN	164	54.7
Education	Secondary education	11	3.7
	Post-secondary education	16	5.3
	Bachelor’s degree	76	25.3
	Master’s degree	197	65.7

Source: the author’s own study.

**Table 3:** Distribution of primary motivating factors among the surveyed nurses.

Motivating Factors	*n*	%
Financial motivating factors (direct financial incentives)		
Financial allowance for overtime/night work	24	8.0
Financial allowances (holiday bonus/vacation allowance)	38	12.7
Incentive bonuses	76	25.3
Salary level	162	54.0
Non-wage motivating factors (indirect material incentives)		
Additional health/pension benefits	36	12.0
Skill development (courses/training at the employer’s expense)	238	79.3
Other (gift cards, reimbursement of commuting costs)	26	8.7
Non-material motivating factors (psychological incentives)		
Friendly relationships with other team members	173	57.67
Flexible working hours	83	27.67
Other (e.g., autonomy/freedom to act, feeling appreciated)	44	14.67

Source: the author’s own study.

**Table 4:** Distribution of primary demotivating factors among the surveyed nurses.

Demotivating Factors	*n*	%
Low autonomy/limited decision-making power	40	13.3
Low occupational status	67	22.3
Low salary/low earnings	146	48.7
Poor relationships with supervisors/the team	47	15.7

Source: the author’s own study.

**Table 5:** Financial Motivating Factors (Direct Monetary Incentives).

Financial Motivating Factors (Direct Financial Incentives)			
Variable	χ^2^	*p*-Value	Cramér’s V
Age	22.505	0.01	0.194
Work seniority	20.162	0.01	0.183
Type of employment	10.036	0.02	0.183
Salary	14.906	0.05	0.158

Source: the author’s own study.

**Table 6:** Non-wage motivating factors (indirect material incentives).

Non-Wage Motivating Factors (Indirect Material Incentives)			
Variable	χ^2^	*p*-Value	Cramér’s V
Age	4.934	0.30	0.091
Work seniority	5.940	0.30	0.099
Type of employment	14.657	0.01	0.221
Salary	4.095	0.50	0.083

Source: the author’s own study.

**Table 7:** Non-material motivating factors.

Non-Material Factors			
Variable	χ^2^	*p*-Value	Cramér’s V
Age	10.157	0.05	0.130
Years of professional experience	13.732	0.01	0.151
Type of employment	5.630	0.06	0.137
Salary	2.409	0.70	0.063

Source: the author’s own study.

**Table 8:** Demotivating factors.

Demotivating Factors			
Variable	χ^2^	*p*-Value	Cramér’s V
Age	11.475	0.10	0.138
Work seniority	14.755	0.05	0.157
Type of employment	0.710	0.90	0.049
Salary	8.629	0.30	0.120

Source: the author’s own study.

## Data Availability

The data supporting the findings of this study are available from the corresponding author upon reasonable request. The data are not publicly available due to privacy and ethical restrictions.

## Supplementary Materials

The following supporting information can be downloaded at: https://www.mdpi.com/article/10.3390/healthcare14172863/s1. Supplementary File S1: Anonymized Research Questionaire.
