# Supplementary material for: Determinants of Nurses’ Work Motivation in the Context of Sustainable Human Resource Management: A Study in a University Clinical Hospital in Poland
Source: Healthcare (Basel). 2026 Sep 5;14(17):2863. doi: 10.3390/healthcare14172863 (PMC13565533; doi:10.3390/healthcare14172863)
Supplement: Supplementary file 1 [file healthcare-14-02863-s001.zip › healthcare-4422536-supplementary.pdf]

SUPPLEMENTARY FILE S1

ANONYMIZED RESEARCH QUESTIONAIRE

SURVEY FOR NURSING STAFF

This questionnaire was used in a research study examining factors influencing work motivation and occupational burnout among nursing staff employed at a university clinical hospital in Poland. Details identifying the institution and the researcher have been removed from this supplementary version.

Participation in the original study was voluntary and anonymous. The collected data were used solely for scientific and research purposes.

Please mark the response that best applies. Where an open response is requested, write on the line provided.

SECTION I. PROFESSIONAL MOTIVATION AND EMPLOYMENT PREFERENCES

1. What motivated you to choose your profession?

- ☐ Conscious choice
- ☐ Unsuccessful recruitment for another field
- ☐ Desire to help others
- ☐ Family example (e.g., mother)

2. Do you feel job satisfaction?

- ☐ Definitely yes
- ☐ Rather yes
- ☐ Rather no
- ☐ Definitely no

3. What motivates you most to work?

- ☐ Financial factors
- ☐ Non-financial factors

4. The most financial motivational factors?

- ☐ Financial allowance for overtime/night work
- ☐ Financial allowances (holiday bonus/vacation allowance)
- ☐ Incentive bonuses
- ☐ Salary level

5. The most non-wage motivational factors?

- ☐ Additional health/pension benefits
- ☐ Skill development (courses/training at the employer's expense)
- ☐ Other (gift cards, reimbursement of commuting costs)

6. The most non-material motivational factors?

- ☐ Friendly relationships with other team members
- ☐ Flexible working hours
- ☐ Other (e.g., autonomy/freedom to act, feeling appreciated)

7. The most demotivating factors at work?

☐ Low autonomy / limited decision-making power
☐ Low occupational status
☐ Low salary / low earnings
☐ Poor relationships with supervisors / the team

8. Do you consider leaving your job?

☐ Definitely yes
☐ Rather yes
☐ Rather no
☐ Definitely no

9. What factors would you consider when looking for a new job?

☐ Job stability
☐ Salary level
☐ Opportunities for development/promotion
☐ Work atmosphere

SECTION II. WORKLOAD, STRESS, AND OCCUPATIONAL EXPERIENCES

10. Do you feel overworked/emotionally exhausted?

☐ Definitely yes
☐ Rather yes
☐ Rather no
☐ Definitely no

11. Is your job a source of stress?

☐ Yes
☐ No

12. The most burdensome aspects of your work?

☐ Too much responsibility
☐ Physical and mental overload
☐ Time pressure

13. Do you feel burned out/frustrated by your work?

☐ Yes
☐ No
☐ I don’t know

14. Have you become more indifferent toward people since starting this job?

☐ Yes
☐ No
☐ I don’t know

15. Do you feel your job has a positive impact on your life?

☐ Yes
☐ No
☐ I don’t know

16. Have you achieved many meaningful goals at work that you are proud of?

☐ Yes
☐ No
☐ I don’t know

SECTION III. SOCIODEMOGRAPHIC AND OCCUPATIONAL CHARACTERISTICS

17. Gender

☐ Female
☐ Male

18. Age

....

19. Work seniority

☐ 0-10 year
☐ 11-20 years
☐ Over 20 years

20. Department/Clinic

....

21. Position

....

22. Type of employment

☐ Permanent employment contract
☐ Fixed-term employment contract

23. Salary level

☐ 3500–5500 PLN
☐ 5600–6500 PLN
☐ 6600–7500 PLN
☐ 7600–8500 PLN
☐ 8600–9500 PLN
☐ Above 10,000 PLN

24. Education completed

☐ Secondary education
☐ Post-secondary education
☐ Bachelor’s degree
☐ Master’s degree
